# How autonomous motivation predicts college students' academic performance: a cross-sectional study on the mediating roles of self-control and learning habits

**DOI:** 10.3389/fpsyg.2026.1771128

**Published:** 2026-03-10

**Authors:** Jihong Xie, Xiaoyong Hu, Penghui Wang, Dan Wang

**Affiliations:** 1 School of Education, Tianshui Normal University, Tianshui, China; 2 Department of Psychology, Wuhan University, Wuhan, China; 3 Counseling Center of Psychology, Tianshui Normal University, Tianshui, China

**Keywords:** academic performance, college students, autonomous motivation, learning habits, self-control

## Abstract

Autonomous motivation can effectively predict students' academic performance; however, the underlying mechanisms through which this occurs require further exploration. Therefore, the purpose of this study is to explore the mediating roles of self-control and learning habits in this relationship among Chinese college students. A cross-sectional survey design was employed. Data were collected via an online questionnaire platform between November and December 2024. Using a convenience sampling method, a total of 796 university students (Mage = 20.49, SD = 1.32) were recruited. Participants completed a series of questionnaires assessing autonomous motivation, self-control, and learning habits. Academic performance scores were also collected. Data analysis was conducted using SPSS 24.0 and the SPSS PROCESS plug-in developed by Hayes. The findings showed that college students' autonomous motivation directly and positively predicted academic performance (effect = 0.072), accounting for 59.02%. Notably, learning habits partially mediated the relationship between autonomous motivation and academic performance (effect = 0.021). In addition, autonomous motivation indirectly and positively affected academic performance through the chain mediating effect of self-control and learning habits (effect = 0.029). The total indirect effect was significant (effect = 0.050), accounting for 40.98% of the total effect. However, the mediating role of self-control alone was not significant. The results of this study elucidate the internal mechanisms linking autonomous motivation to academic performance and provide actionable insights for college teachers and educational departments to improve students' academic outcomes.

## 1 Introduction

Academic performance is a crucial indicator for evaluating students' learning outcomes across all levels and types of education. It also serves as an important reference for determining whether students meet the requirements of their program (Namoun and Alshanqiti, 2020). Moreover, academic performance is closely related to future job performance (Kuncel et al., 2005) and students' persistence in learning (Ruban and McCoach, 2005). It is influenced by multiple factors, including intelligence (Roth et al., 2015), personality (Poropat, 2009), social class (Damian et al., 2015), and motivation (Kriegbaum et al., 2018).

Traditionally, students with strong learning motivation are believed to achieve higher academic performance (Sabanal et al., 2023). In addition, some studies suggest that adolescents' and young adults' motivation for learning and working tends to increase with age, especially during the transition period before entering the workforce. This stage is extremely important for developing motivation, highlighting the need for studying how college students' learning motivation impacts their academic performance (Symonds et al., 2019). A substantial body of research indicates that learning motivation directly and positively predicts academic achievement (Boncquet et al., 2020; Jeroen et al., 2022; Jeno et al., 2018; Li et al., 2019). However, these studies have not explored the specific pathways through which learning motivation affects academic performance. To address these gaps, this paper explores the underlying mechanisms through which college students' autonomous motivation predicts academic performance.

## 2 Literature review and research hypotheses

### 2.1 Autonomous motivation and academic performance

Motivation drives individuals' behavioral activities, inspiring and sustaining people's efforts to achieve specific goals. Motivation explains a significant portion of the variance in academic achievement, even after accounting for intelligence (IQ) and personality (Jeroen et al., 2022). Traditional theories focus on the strength or quantity of motivation, while self-determination theory distinguishes between autonomous motivation and controlled motivation. The former arises from individuals' genuine interests or their own will, while the latter is driven by internal or external pressures and the pursuit of rewards, honor, or prestige (Ryan and Deci, 2020).

In the context of student learning, autonomous motivation refers to the drive that arises from students' inherent volition (Ryan and Deci, 2017; Kappes et al., 2023). It encompasses three categories: (1) Intrinsic motivation, in which students engage in learning because they find the content inherently interesting, enjoyable, or challenging; (2) integrated regulation, which reflects the alignment of learning behaviors with students' self-identity and personal values; and (3) identified regulation, whereby students recognize the personal significance or relevance of learning and thus engage willingly (Mouratidis et al., 2021).

In contrast, controlled motivation refers to learning driven by internal or external psychological pressures (Ryan and Deci, 2017). It includes introjected regulation, in which students study to avoid feelings of guilt or shame or to maintain self-worth, and external regulation, in which learning is primarily motivated by the desire to obtain external rewards or avoid punishments from teachers or parents. Overall, the quality of motivation is a key determinant of learning outcomes (Mouratidis et al., 2021), with high-quality motivation characterized by high levels of autonomous motivation combined with low levels of controlled motivation.

Previous studies have shown that autonomous motivation, compared to controlled motivation, predicts better outcomes in multiple areas such as education, health behaviors, and interpersonal relationships (Benita et al., 2023; Ryan and Deci, 2020; Ntoumanis et al., 2021; Legault and Amiot, 2014). For example, a study involving undergraduate and postgraduate students found that autonomous motivation significantly and positively predicted academic performance, as measured by final-semester biology exam scores (Jeno et al., 2018). Similarly, a study on junior high school students reported that autonomous motivation significantly and positively predicted students' academic performance and satisfaction with school (Li et al., 2019). As well, the results of the latent variable change model in a longitudinal research study indicated that primary school students with higher autonomous motivation not only achieved better math scores but also showed greater progress in math during the transition to middle school (Boncquet et al., 2020). These findings highlight that autonomous motivation has a significant impact on academic performance. Therefore, this study proposes the following hypothesis:

Autonomous motivation significantly and positively predicts college students' academic performance (*Hypothesis 1*).

As already highlighted, autonomous motivation has a significant positive predictive effect on academic performance. However, the mechanisms through which autonomous motivation predicts academic performance require further exploration. (Aydin and Michou 2020) suggested that college students' self-determined motivation predicts academic performance through the mediating role of academic buoyancy. A similar study found that motivation traits could predict academic performance in high school students through the mediating role of achievement goals (Dickhäuser et al., 2016). However, the mediating variables in the relationship between autonomous motivation and academic performance are not limited to academic buoyancy or achievement goals. Other factors, such as self-control and learning habits, may also play important roles in this process.

### 2.2 The mediating role of self-control

Self-control refers to an individual's ability to manage and regulate attention, cognition, emotions, and behaviors, particularly when high-value long-term goals conflict with low-value short-term goals. Individuals with higher levels of self-control demonstrate a stronger tendency to approach their goals in an autonomous manner. Within Self-Determination Theory (Deci and Ryan, 2000), autonomous motivation refers to pursuing goals because individuals “want to,” rather than because they “have to.” This relationship exhibits an instrumental character, as evidenced by its heightened salience during active goal pursuit compared to after goal completion (Converse et al., 2019). Conversely, when actions are driven by external pressures or rewards, individuals exhibit reduced task persistence and impaired performance in subsequent activities (Deci et al., 1999; Bénabou and Tirole, 2003).

Individuals' autonomous motivation may mitigate the depletion of self-control resources and enhance subsequent proper functioning (Dou et al., 2017; Laran and Janiszewski, 2011). Recent studies also indicate that autonomous motivation significantly plays a crucial role in self-control (Steel et al., 2021).

Self-control is also closely related to academic achievement (Tangney et al., 2004; Zhou and Bi, 2017). It can predict academic performance across various educational stages, including primary, secondary, and university education, as well as standardized achievement test scores (Duckworth et al., 2019). A 2 year follow-up study found that self-control significantly predicted teacher-reported academic competence and school-reported grades (Vazsonyi et al., 2022). Some scholars even argue that self-control is a stronger predictor of academic performance than intelligence (Duckworth and Seligman, 2005).

Existing research similarly indicates that self-control is a key mediating mechanism explaining the differential impacts of various motivational types on wellbeing (Zeng and Chen, 2020). Specifically, fulfillment motivation emphasizes the pursuit of long-term goals and intrinsic values, which align closely with individuals' core value orientations. Consequently, when facing conflicts between short-term desires and long-term objectives, individuals driven by fulfillment motivation are more likely to mobilize higher levels of self-control to achieve their goals (Huta, 2015; Sheldon, 2014). Fulfillment motivation shares certain similarities with the autonomous motivation examined in this study. Attaining favorable academic performance typically requires sustained, long-term effort, during which students must employ self-control to overcome short-term desires—such as entertainment and social activities—that conflict with learning behaviors. Based on these research results, this study posits the following hypothesis:

Autonomous motivation may predict college students' academic performance through the mediating role of self-control (*Hypothesis 2*).

### 2.3 The mediating role of learning habits

Habits are automatic behaviors or response tendencies triggered by situational cues (Lally et al., 2010; Wood and Neal, 2007). Learning habits, specifically, are automatic behaviors and tendencies related to learning that are triggered by situational cues. These habits are very important as they contribute to the development of knowledge, perceptual abilities, and academic performance (Rabia et al., 2017). For example, research by (Bibi et al. 2020) noted that high school students with high academic performance also exhibited better learning habits. Better learning habits, such as recalling recently memorized information and preferring the information to be presented in a graphical form, are closely linked to academic achievement in a study involving medical university students (Aljaffer et al., 2024). Similarly, another study on middle school students also highlighted how reading habits specifically can significantly predict academic performance (Abid et al., 2023). This research supports the idea that college students' learning habits may also predict their academic performance.

Moreover, the formation of learning habits is closely related to individuals' autonomous motivation for learning. A person who is motivated and enjoys learning will more likely study frequently or participate in various learning-related activities, gradually developing good learning habits. The goal-habit interaction model holds that everyday habits develop as people pursue their life goals, such that habit formation is closely intertwined with goal pursuit and goal-directed behavior (Wood and Rünger, 2016). Such goal-directed behaviors depend on motivation to inspire and maintain them. Moreover, studies on the relationship between habits and autonomous motivation show that autonomous motivation is more strongly associated with automatic behaviors than non-autonomous motivation (Radel et al., 2017). A cross-lagged analysis study on college students revealed that there was a significant correlation between physical activity habits and autonomous motivation (Phipps et al., 2023). Based on these findings, this study proposes the following hypothesis:

Learning habits play a mediating role in the relationship between autonomous motivation and college students' academic performance (*Hypothesis 3*).

### 2.4 The chain mediating roles of self-control and learning habits

The previous discussion has illustrated the separate mediating roles of self-control and learning habits in the relationship between college students' academic performance and autonomous motivation. In fact, self-control and behavioral habits are closely linked. People with high self-control are more inclined to overcome negative habits and maintain positive ones in daily life. For example, one study asked college students to record their unhealthy snack consumption in a 7 day diary and then measured their self-control levels. The results showed that those with higher self-control had fewer unhealthy eating habits (Adriaanse et al., 2014). Self-control can also significantly predict beneficial habits. A study involving adolescents and middle-aged individuals examined multiple behavioral habits, including physical exercise, diet, sleep, homework completion, and study habits. It found that self-control ability may influence these different habits (Galla and Duckworth, 2015). Other similar investigations also demonstrated that self-control might be more closely tied to the development of adaptive habits than to situational impulse regulation (De Ridder et al., 2012). In essence, the advantage of highly self-disciplined individuals lies in this: they struggle to form habits around unhealthy activities, such as eating junk food (Adriaanse et al., 2014), while easily establishing solid habits for healthy ones like sleep, exercise, and work (Galla and Duckworth, 2015; Gillebaart and Adriaanse, 2017). As previously discussed, autonomous motivation may predict self-control, and learning habits may predict academic performance. Based on these insights, this study proposes the following hypothesis:

Self-control and learning habits mediate the relationship between autonomous motivation and college students' academic performance (*Hypothesis 4*).

**Figure 1:**
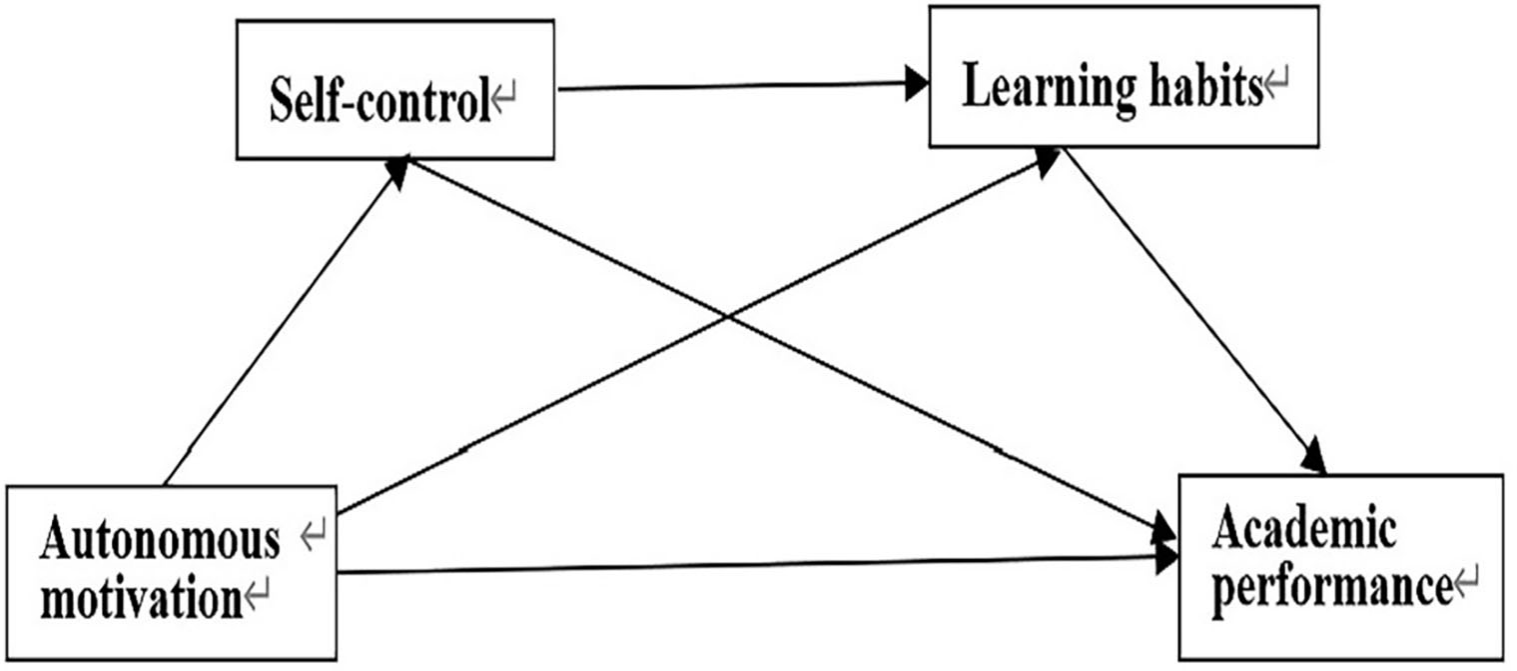
Hypothetical mediating effect model.

Building on the four hypotheses discussed above, this study proposes a mediating model in which autonomous motivation predicts college students' academic performance, as shown in Figure 1.

## 3 Methods

### 3.1 Participants and data collection

Participants were recruited from Tianshui Normal University in Gansu Province, China. A convenience sampling method was employed. All participants were full-time undergraduate students in their first to third years of study. Fourth-year students were excluded due to the unique circumstances they typically face, such as intensive focus on thesis writing and job-seeking activities, alongside the fact that most majors have largely completed their exam-based coursework by this stage. A total of 899 questionnaires were distributed. After excluding 103 electronic questionnaires with excessive missing answers, 796 valid forms were obtained. The effective recovery rate was 88.5%. Participants' ages ranged from 17 to 25 years old, with an average age of 20.49 years (SD = 1.32). The sample comprised 139 males (17.5%) and 657 females (82.5%). In terms of academic year, 201 participants (26.3%) were first-year, 303 (38.1%) were second-year, and 284 (35.7%) were third-year university students. The participants represented a range of academic disciplines, including Education (10.1%), History (6.9%), Ideological and Political Education (23.2%), Literature (2.8%), English (8.7%), Mathematics (4.8%), Accounting (14.8%), Mechanical Engineering (5.5%), Biological Sciences (17.7%), and Civil Engineering (5.5%).

### 3.2 Study design and procedure

The present study design was reviewed and approved by the Ethics Committee of the School of Education of Tianshui Normal University. Data were collected via an online questionnaire survey using the Questionnaire Star platform. The data collection period spanned from November to December 2024. Participants accessed the survey page via a designated link, which contained the informed consent form, a brief introduction to the study, and the measurement instruments (see the “Measures” section below). Prior to completing the questionnaire, participants were informed that: (a) the study aimed to examine the relationship between daily learning activities and academic achievement; (b) eligibility for participation required regular attendance in course activities and final examinations during the current semester; and (c) all questionnaire data would be completely anonymous, and participants were encouraged to provide truthful responses. Upon completion of the questionnaire, participants were provided an opportunity to enter a prize draw as compensation for their participation.

### 3.3 Measures

#### 3.3.1 Autonomous motivation questionnaire

The Autonomous Motivation Questionnaire (AMQ) was created by the author based on the Self-Regulation Style Questionnaire-Academic (SRQ-A) developed by (Ryan and Connell 1989), with reference to its Chinese revised version (Liu et al., 2013). Participants were first asked about their reasons for engaging in specific learning behaviors and then selected responses representing different motivation types. For example, one question asked, “I want to study my major courses hard because...,” and each option presented a reason based on motivation type: “because others want me to do it, or other external reasons” (external regulation); “if I don't do it, I will feel guilty, ashamed, or anxious” (introjected regulation); “because I think this thing is really important to me” (identified regulation); or “because I find it interesting, it's a pleasure or even an enjoyment for me” (intrinsic regulation). Participants rated each item on a 5-point scale, from 1-very inconsistent to 5-very consistent. Items corresponding to the same motivation type were combined to form subscales (Ryan and Connell, 1989).

Participants responded to three learning-related questions, each associated with four possible responses grounded in the aforementioned types of regulation (external, introjected, identified, intrinsic), resulting in a total of 12 options. The internal consistency coefficient α was 0.800, with subscale α values of 0.878, 0.910, 0.790, and 0.841 for external regulation, introjected regulation, identified regulation, and intrinsic regulation, respectively. The autonomous motivation index was calculated as follows: the identified regulation scores plus the intrinsic regulation scores of the three learning behavior reasons, minus the introjected regulation and external regulation scores. Higher scores indicated a greater tendency toward autonomous motivation (Williams et al., 1996).

#### 3.3.2 Self-control scale

This study adopted the Chinese revised version of the Self-Control Scale (Tan and Guo, 2008). This scale consists of 19 items, with an internal consistency coefficient α of 0.874. It contains five dimensions: impulse control, healthy habits, resistance to temptation, concentration on work, and moderation of entertainment. A 5-point scoring method was used, from 1 - very inconsistent to 5 - very consistent. Once again, the higher the score, the higher the level of self-control.

#### 3.3.3 Learning habits questionnaire

Habits refer to the repeated occurrence of a certain behavior in situations with relatively consistent characteristics, such as location and time of behavior occurrence. Drawing on the learning habit strength measurement method proposed by (Galla and Duckworth 2015), three self-reported items were used in this research to measure participants' learning habits over the past 6 months. The first item measured the frequency of learning behavior occurrence (except for regular classes), rated as: 1 = I hardly study every week; 2 = I occasionally study every week; 3 = I study about half of the time every week; 4 = I study most of the time every week; 5 = I study every day, every week. The second and third items measured the degree of fixation of the location (e.g., “I tend to study in a fixed location”) and time (e.g., “I tend to study at regular times”), respectively. Participants were asked to rate each item on a 5-point scale from 1-very inconsistent to 5-very consistent. The total score, obtained by adding the three items, was used to form a learning habit strength index. In this questionnaire, the higher scores represented stronger learning habits. The coefficient α of the three items was 0.625.

#### 3.3.4 Academic performance

Academic performance was assessed using the average course scores at the end of the first semester of the 2024-2025 academic year. After the final exams, the researchers collaborated with course instructors to obtain the average final scores for each student. To align students' academic performance data with the questionnaire responses, participants were asked to provide their student ID (identity) information when completing the questionnaires. After obtaining the learning performance data, the final course scores for each individual were matched with the corresponding questionnaire data.

### 3.4 Data analysis

After collecting the questionnaires and academic performance scores, SPSS 24.0 was used to conduct descriptive statistics for each variable and perform correlation analysis among autonomous motivation, self-control, learning habits, and academic performance. Mediation analysis was then performed using Model 6 of the SPSS PROCESS macro (version 4.2) developed by (Hayes 2013), enabling the assessment of both the direct and indirect effects of autonomous motivation, self-control, and learning habits on academic performance.

## 4 Results

### 4.1 Common-method bias test

In this study, autonomous motivation, self-control, and learning habits were measured through self-assessment, which could lead to common method bias. However, data collection methods were employed to help reduce the risk. First, the outcome variable—academic performance—is an objective test result, fundamentally different from the self-reported predictors, reducing the likelihood of method bias. Second, data were collected online: students accessed the questionnaire via links shared by teachers in class WeChat groups and could respond at their own convenience. This decentralized process minimized situational consistency and classroom-based administration effects. Third, the questionnaires used for measuring autonomous motivation, self-control, and learning habits differed structurally, further limiting method-based bias.

The Harman single-factor test was conducted. The test results showed seven factors with eigenvalues greater than 1. The percentage of variance explained by the factor with the largest eigenvalue was 21.2%, below the commonly recognized threshold of 40%. This indicates that there was no serious common method bias.

### 4.2 Descriptive statistics and correlation analysis of variables

The means, standard deviations, and correlation coefficients for the four variables in the study are presented in Table 1. There were significant positive correlations between academic performance, autonomous motivation, self-control, and learning habits.

**Table 1:** Correlations of the four main variables (*N* = 950).

**Variable**	**M**	**SD**	**1**	**2**	**3**	**4**
1Academic performance	80.64	6.33	1			
2 Self-control	63.97	10.24	0.11^**^	1		
3 Study habits	10.39	2.08	0.23^**^	0.40^**^	1	
4 Autonomous motivation	6.23	6.65	0.13^**^	0.41^**^	0.25^**^	1

^**^p < 0.01.

### 4.3 Chain Mediating Roles of Self-control and Learning Habits

This study constructed a chain mediation model with autonomous motivation as the independent variable, academic performance as the dependent variable, and self-control and learning habits as mediating variables. Data analysis was conducted using Model 6 of Hayes' PROCESS macro for SPSS, with 5,000 bootstrap resamples at 95% confidence interval. The results are summarized in Table 2.

**Table 2:** Regression analysis of the mediating model.

Regression equation		Overall fit index			Coefficients and significance	
**Result variables**	**Predictive variables**	***R***	***R** ^2^*	***F***	β	***t***
Self-control	Autonomous motivation	0.414	0.172	86.913	0.414	12.821^**^
Learning habits	Autonomous motivation	0.412	0.169	80.881	0.107	2.999^**^
	Self-control				0.356	10.008^**^
Academic performance	Autonomous motivation	0.237	0.056	15.669	0.076	1.995^*^
	Self-control				0.0003	0.007
	Learning habits				0.206	5.424^**^

Coefficients are standardized; bootstrap samples = 5,000; ^**^p < 0.01, ^*^p < 0.05.

**Figure 2:**
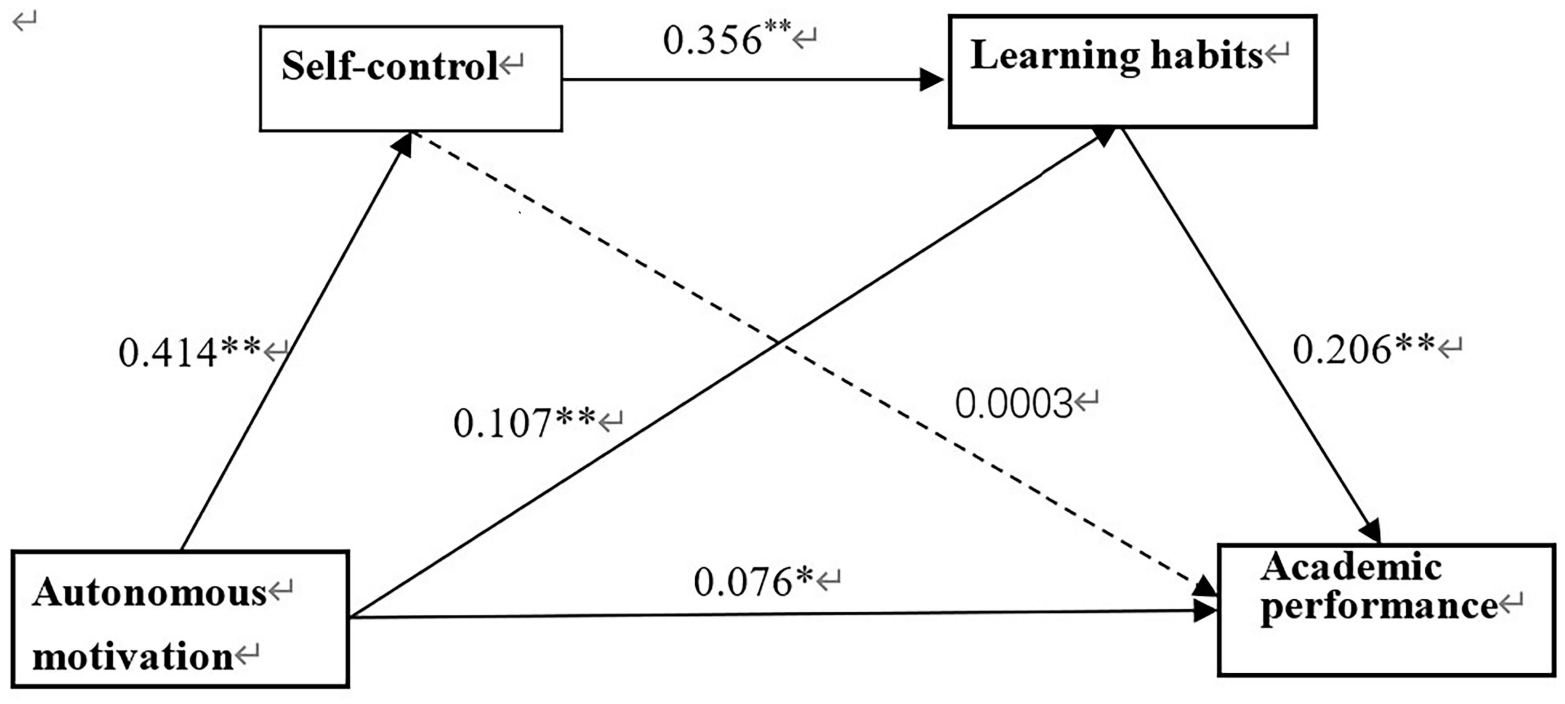
Mediating effects model.

When autonomous motivation, self-control, and learning habits were entered into the regression equation simultaneously, the direct positive predictive effect of autonomous motivation on academic performance was significant (β = 0.076, *P* < 0.05), while the predictive effect of self-control on academic performance was not significant (β = 0.0003, *P* > 0.05). Moreover, the positive predictive effect of learning habits on academic performance was significant (β = 0.206, *P* < 0.01). At the same time, autonomous motivation significantly and positively predicted self-control (β = 0.414, *P* < 0.01) and learning habits (β = 0.107, *P* < 0.01). Self-control also significantly and positively predicted learning habits (β = 0.356, *P* < 0.01). The results are shown in Figure 2.

Model 6 of the SPSS PROCESS (version 4.2) plug-in developed by Hayes was used to analyze the effects of the research variables, with the bias-corrected Bootstrap method applied for testing. The results are shown in Table 3.

**Table 3:** The direct and indirect effects of the model.

**Effect type**	**Effects**	**Boot SE**	**Boot LLCI**	**Boot ULCI**	**Effect percentage**
Direct effect	0.072	0.036	0.001	0.144	59.02%
Total indirect effect	0.050	0.020	0.014	0.092	40.98%
Indirect effect 1	0.000	0.026	−0.044	0.059	0.00%
Indirect effect 2	0.021	0.010	0.005	0.044	17.21%
Indirect effect 3	0.029	0.009	0.012	0.049	23.77%

SE, LLCI, and ULCI refer to the standard error and the upper and lower bounds of the 95% confidence intervals of the indirect effects estimated by the bootstrap method, respectively. Indirect effect 1: Autonomous motivation → Self-control → Academic performance; Indirect effect 2: Autonomous motivation → Learning habits → Academic performance; Indirect effect 3: Autonomous motivation → Self-control → Learning habits → Academic performance.

The analysis revealed that college students' autonomous motivation significantly and directly predicted their academic performance (effect = 0.072, 95% CI: 0.001 to 0.144), accounting for 59.02% of the total effect. Learning habits played a partial mediating role in this relationship. The mediating effect test results indicated that the partial mediating effects of learning habits were significant. The total indirect effect value was significant (effect = 0.050, 95% CI: 0.014 to 0.092), accounting for 40.98% of the total effect. Specifically, the mediation occurred through two paths: 1) autonomous motivation → learning habits → academic performance (effect2 = 0.021, 95% CI: 0.005 to 0.044), accounting for 17.21% of the total effect; and 2) autonomous motivation → self-control → learning habits → academic performance (effect3 = 0.029, 95% CI: 0.012 to 0.049), accounting for 23.77% of the total effect. However, the partial mediating effect of self-control in the prediction of academic performance by autonomous motivation was not significant (effect1 = 0.000, 95% CI:−0.044 to 0.059).

## 5 Discussion

### 5.1 Autonomous motivation as a predictor of academic performance

This study found that autonomous motivation significantly and positively predicted college students' academic performance, verifying Hypothesis 1. This is consistent with the results of many previous studies (Froiland and Oros, 2014; Richardson et al., 2012). According to self-determination theory, autonomous motivation facilitates a range of beneficial individual outcomes (Ryan and Deci, 2020). Under the influence of autonomous motivation, people more willingly engage in work tasks aligned with their personal interests, which makes them extremely appealing, providing a sense of happiness and satisfaction. In such cases, people can more effectively activate their internal energy, leading to greater efficiency in their work efforts. Similarly, learning behaviors driven by autonomous motivation originate from individuals' personal desires and choices will show more investment and persistence in learning activities, and ultimately, higher-quality learning results (Ryan and Deci, 2000).

### 5.2 The mediating role of self-control

The results of this study showed that although college students' autonomous motivation significantly predicted self-control (β = 0.414, *P* < 0.01), the direct path from self-control to academic performance was not significant (β = 0.0003, *P* > 0.05). This indicates that self-control did not mediate the relationship, and Hypothesis 2 of this study was not verified. This finding aligns with previous research on the predictive relationship between these two variables (Dzinović et al., 2019).

This study provides evidence that autonomous motivation has a significant positive predictive effect on self-control. According to Self-Determination Theory, autonomous motivation stems from individuals' intrinsic identification with and integration of the value of their actions, which helps mobilize students' internal resources and enhance self-regulatory capacity during goal pursuit (Deci and Ryan, 2000). Specifically, higher levels of autonomous motivation may reduce the occurrence of “action crises,” defined as the motivational conflicts between persisting with or abandoning a goal in the face of setbacks (Holding et al., 2017). In academic contexts, autonomous motivation not only serves as a key driver of academic progress but also supports adaptive self-regulatory processes, such as goal adjustment and emotion management (Howard et al., 2021). In summary, by satisfying the need for autonomy and optimizing self-regulation, autonomous motivation enhances self-control, which is consistent with the theoretical expectations of Self-Determination Theory.

However, this study found that self-control did not significantly predict academic achievement. Several explanations can be proposed for this finding. First, the process model of self-control posits that self-control consists of multiple, distinct strategic regulation processes rather than a single, independent psychological or behavioral response (Inzlicht et al., 2021; Duckworth et al., 2016). Therefore, when it is associated with another variable, for example, academic performance, intermediate variables may be involved. For example, when academic self-efficacy is included as a mediating variable, self-control significantly predicts academic achievement (Dzinović et al., 2019). Second, from the perspective of the predictive effect of self-control on academic performance, achieving higher academic performance requires continuous self-control behaviors. However, continuous self-control requires overcoming inherent challenges, such as avoiding motivation conflicts and resource depletion (Uziel, 2018). This indicates that students may only achieve superior academic performance when their self-control abilities are translated into effective self-regulation strategies (Vazsonyi et al., 2022). Alternatively, through sustained self-control, students may develop effective learning habits, whereby self-control could indirectly enhance academic achievement via the mediating role of such habits. This constitutes a key finding of the present study. Therefore, the impact of self-control on academic performance is unlikely to manifest as a direct effect. Considering intermediate variables provides a better comprehension of how self-control predicts academic performance.

### 5.3 The mediating role of learning habits

This research reported that college students' autonomous motivation for learning significantly and positively predicted their learning habits, which, in turn, predicted their academic performance. Moreover, learning habits played a partial mediating role between autonomous motivation and academic performance, verifying Hypothesis 3. The mediating mechanism identified in this study diverges from those documented in prior literature. Previous studies have shown that intrinsic motivation can indirectly improve academic performance by boosting self-efficacy (Shen et al., 2025), influence academic achievement through online learning behaviors (Meng and Hu, 2023), or exert a significant indirect effect on academic outcomes via enhanced learning engagement (Wu et al., 2020). Given the conceptual affinity between intrinsic motivation and the autonomous motivation examined in this study, autonomous motivation is also likely to affect academic achievement through multiple pathways. The present findings further suggest that study habits serve as a crucial mediating variable in the relationship between autonomous motivation and academic performance.

These results align with those of prior studies showing that autonomous motivation plays an important role in the formation of behavioral habits (Gardner and Lally, 2013). From the perspective of Self-Determination Theory, autonomous motivation arises from individuals‘ intrinsic interest in and identification with the value of their behaviors, and is often experienced as enjoyment during goal pursuit (Benita et al., 2023). This motivational trait reflects proactive awareness and a voluntary, self-initiated behavioral tendency, thereby promoting more frequent and greater engagement in goal-directed activities. Moreover, autonomous motivation promotes the automaticity of behavior, leading individuals to act with minimal conscious deliberation (Ryan and Deci, 2000). For college students with autonomous motivation for learning, their learning behaviors are spontaneously generated. These behaviors, carried out repeatedly at specific times and places, become habitual responses that do not require external intervention. Good learning habits, such as continuously reading and doing homework at fixed times and places, enable students to understand and process knowledge more frequently and deeply, leading to greater mastery of the learning content. As a result, college students' academic performance will naturally improve over time due to good learning habits.

### 5.4 The chain mediating roles of self-control and learning habits

Another important finding of this study is that a mechanism through which autonomous motivation predicts academic performance is achieved through the chain mediating roles of self-control and learning habits, verifying Hypothesis 4. The serial mediation pathway identified in this study represents a distinct mechanism compared to those reported in prior research. For instance, intrinsic motivation has been found to influence medical students' academic performance through the sequential mediation of self-efficacy and learning engagement (Wu et al., 2020). In another study involving low-income undergraduates, academic achievement was affected by intrinsic motivation via two parallel mediators: study strategies and perceived stress (Huang et al., 2025). While these studies, like the present one, examine motivation as a predictor of academic outcomes, both the mediators and the pathway structures differ. Specifically, our findings demonstrate that autonomous motivation enhances academic performance through a serial mediation chain consisting of self-control and learning habits.

Specifically, the process by which autonomous motivation affects academic performance happens through three linked paths: autonomous motivation predicts self-control, self-control predicts learning habits, and learning habits predict academic performance.

First, autonomous motivation predicts self-control. Learning activities triggered by autonomous motivation are intrinsically interesting and meaningful to students, making them more likely to believe in their ability to succeed. They are also more inclined to believe that they can study effectively, rather than admitting difficulty in focusing on learning (Converse et al., 2019). Students with this mindset exhibit stronger autonomy and self-discipline in their learning behaviors, actively overcoming various distractions and challenges during the learning process, and flexibly adjusting their goal pathways—rather than rigidly persisting—when encountering obstacles during goal pursuit (Kappes et al., 2023; Holding et al., 2022). As a result, autonomous motivation for learning stimulates self-control behaviors during the learning process. The self-energy vitality model also points out that autonomous motivation can affect the self-control tasks individuals engage in (Ryan and Deci 2008); (Song et al. 2015). This aligns with the broader understanding of general motivation's role in self-control: when motivation is lacking, self-control may fail, but when motivation is strong, self-control may persist even in a depleted state (Yu et al., 2013). Second, this study suggests that college students with higher self-control abilities are more likely to have stronger study habits. This aligns with previous research, which has shown that individuals with high self-control often have many positive and healthy behavioral habits, such as physical exercise, work, and learning (Carden and Wood, 2018). Third, learning habits predict academic performance. This may be because learning habits can weaken conflicts between learning and distractions, such as reducing intrusive thoughts, negative emotions, and inappropriate learning behaviors (Galla and Duckworth, 2015). Thus, high-intensity learning habits provide a solid foundation for effective learning, significantly and positively predicting academic performance.

In summary, grounded in Self-Determination Theory, autonomous motivation in academic contexts enables students to mobilize personal resources and regulatory strategies more effectively, including self-regulation skills and the formation of productive learning habits, thereby exerting a positive influence on academic performance. Hence, supporting the development of students' autonomous motivation, enhancing self-control capacity, and fostering effective learning habits are of significant importance for promoting adaptive learning behaviors and sustainable academic growth.

### 5.5 The small effect sizes and low model *R*^2^

The analysis revealed a small effect of autonomous motivation on academic achievement (see Table 3). One possible explanation is that academic performance is shaped by a wide array of interrelated factors (Duckworth et al., 2019). The variables examined in this study—autonomous motivation, self-control, and learning habits—therefore represent only a subset of the factors that influence academic outcomes. Previous research has indicated that complex psychological phenomena, such as cognitive ability, are typically determined by multiple factors, with each individual factor often exerting only a modest independent effect (Götz et al., 2022). Furthermore, the magnitude of an effect size must be interpreted within its specific context; effect sizes lack meaningful significance when divorced from appropriate frames of reference (Funder and Ozer, 2019).

From a practical perspective, although the effects of autonomous motivation, self-control, and study habits on academic performance are small, consistent maintenance of high levels of these factors over time may lead to significant improvements in academic outcomes. In research on individual differences, such as personality traits, even traits with minimal influence on a single behavior can cumulatively affect important life outcomes, including personal achievement (Funder and Ozer, 2019). Additionally, slight overall mean changes may mask significant shifts at the distribution extremes (Carey et al., 2023). For instance, widespread educational interventions targeting students with low autonomous motivation and poor study habits could produce notable overall enhancement of academic performance at the school or even national level, as small effects accumulate over time and at scale (Funder and Ozer, 2019; Götz et al., 2022).

The regression models in this study yielded relatively low coefficients of determination (see Table 2). Specifically, when autonomous motivation was included as the initial predictor, it accounted for only 5.6 % of the variance in academic achievement (*R*^2^ = 0.056), suggesting limited predictive power in this context. This outcome likely stems from several factors. First, academic achievement is inherently shaped by a multitude of influences, as noted previously. Second, cross-cultural differences in motivational patterns may attenuate the predictive role of autonomous motivation. For instance, in U.S. settings, science-related self-concept is often the strongest predictor of achievement, whereas in Singapore, the utility value of science learning is more dominant (Zhang et al., 2023). These differences reflect broader cultural orientations: Western, individualistic cultures emphasize self-belief and intrinsic drives, which align closely with autonomous motivation and directly foster student engagement and performance. By contrast, East Asian societies, including Singapore and China, often emphasize the social and pragmatic value of learning, a tendency reinforced by Confucian educational traditions that frame learning as a means of fulfilling social and familial expectations (Tweed and Lehman, 2002; Markus and Kitayama, 1991). In such settings, learning is often perceived as a vehicle for future academic and career advancement, with utility value serving as the primary motivator. Accordingly, students' motivations are frequently socially oriented (e.g., gaining respect or meeting community standards) (Chang and Wong, 2008; van Egmond et al., 2013) and are typically more instrumental and externally regulated. Given that these prevalent motivational forms diverge substantially from the construct of autonomous motivation examined in this study, it is plausible that autonomous motivation exerts a weaker influence on academic achievement in East Asian educational environments.

## 6 Conclusion

This study reveals the important roles that self-control and learning habits play when autonomous motivation predicts college students' academic performance. It demonstrates that autonomous motivation not only directly and significantly predicts academic performance but also significantly and positively predicts academic performance through the mediating role of learning habits and the chain mediating roles of self-control and learning habits. These research results confirm the importance of self-control and learning habits in college students' learning activities and offer insights for teachers and education departments on how to improve students' academic performance through autonomous motivation.

## 7 Limitations and implications

### 7.1 Limitations

There were some limitations in this study. First, the research subjects were college students from one province in mainland China, which may impact the representativeness of the sample. Therefore, the ecological validity of the research conclusions is limited, and further research should explore these research conclusions across different population groups. Second, this study employed a cross-sectional design, making it difficult to establish causal relationships between variables. Subsequent related research should consider the longitudinal tracking method. Third, it was difficult to avoid the subjective bias of participants when answering questionnaires. Convenience sampling is prone to selection bias (Etikan Musa and Alkassim, 2016), and the representativeness of the sample to the target population requires further scrutiny. More rigorous sampling methods are warranted in replication studies of a similar nature. Therefore, caution is advised when generalizing the findings of the present study.

In this study, the internal consistency coefficient (α) of the learning habits questionnaire was relatively low, reflecting limitations in the measurement instrument and survey process. Additionally, the correlation coefficients between the study variables and academic performance were modest, which is consistent with the small effect sizes and low coefficient of determination (*R*^2^) observed in this study. These results collectively indicate that the predictors examined account for a relatively small proportion of the variance in academic performance. Therefore, the findings should be interpreted with caution in practical contexts.

### 7.2 Implications

This study found that autonomous motivation predicts academic performance through two indirect paths: one through the independent mediating role of learning habits, and the other through the chain mediating role of self-control and learning habits. These research results clarify the specific psychological process through which autonomous motivation predicts academic performance, revealing the internal mechanisms at play and providing a new perspective for understanding how autonomous motivation predicts academic performance.

In educational practice, understanding the mediating roles of self-control and learning habits in the relationship between autonomous motivation and academic performance offers valuable insights for educators. Colleges can take steps aimed at enhancing students' autonomous motivation, such as providing more autonomy in course selection and project topics. Simultaneously, fostering self-control skills through time management workshops and promoting the formation of healthy learning habits via study skills training could help create a more conducive environment for academic success. By targeting these three elements in combination, institutions can help students unlock their full academic potential.

## Data Availability

The datasets presented in this study can be found in online repositories. The names of the repository/repositories and accession number(s) can be found in the article/supplementary material.
